# Supplementary material for: High-throughput screening unveils nitazoxanide as a potent PRRSV inhibitor by targeting NMRAL1
Source: Nat Commun. 2024 Jun 6;15:4813. doi: 10.1038/s41467-024-48807-y (PMC11156899; doi:10.1038/s41467-024-48807-y)
Supplement: Supplementary file 1 — Supplementary Information [file 41467_2024_48807_MOESM1_ESM.pdf]

Supplementary Information

High-Throughput Screening Unveils Nitazoxanide as a Potent PRRSV Inhibitor by Targeting NMRAL1

Zhanding Cui, Jinlong Liu, Chong Xie, Tao Wang, Pu Sun, Jinlong Wang, Jiaoyang Li, Guoxiu Li, Jicheng Qiu, Ying Zhang, Dengliang Li, Ying Sun, Juanbin Ying, Kun Li, Zhixun Zhao, Hong Yuan, Xingwen Bai, Xueqing Ma, Pinghua Li, Yuanfang Fu, Huifang Bao, Dong Li, Qiang Zhang, Zaixin Liu, Yimei Cao*, Jing Zhang*, Zengjun Lu*

* Corresponding authors.
Email: Zhanding Cui, E-mail: tata989837@gmail.com, Yimei Cao, E-mail: caoyimei@caas.cn, Jing Zhang, E-mail: zhangjing@caas.cn, Zengjun Lu, E-mail: luzengjun@caas.cn

This file includes:

Supplementary Figs. 1 to 14
Supplementary Tables 1 to 4

Content list

Supplementary Figures

Supplementary Fig. 1 -- 1
Supplementary Fig. 2 -- 2
Supplementary Fig. 3 -- 3
Supplementary Fig. 4 -- 4
Supplementary Fig. 5 -- 5
Supplementary Fig. 6 -- 6
Supplementary Fig. 7 -- 7
Supplementary Fig. 8 -- 8
Supplementary Fig. 9 -- 9
Supplementary Fig. 10 -- 10
Supplementary Fig. 11 -- 11
Supplementary Fig. 12 -- 12
Supplementary Fig. 13 -- 13
Supplementary Fig. 14 -- 14

Supplementary Tables

Supplementary Table 1 --- 15
Supplementary Table 2 --- 16
Supplementary Table 3 --- 17
Supplementary Table 4 --- 17

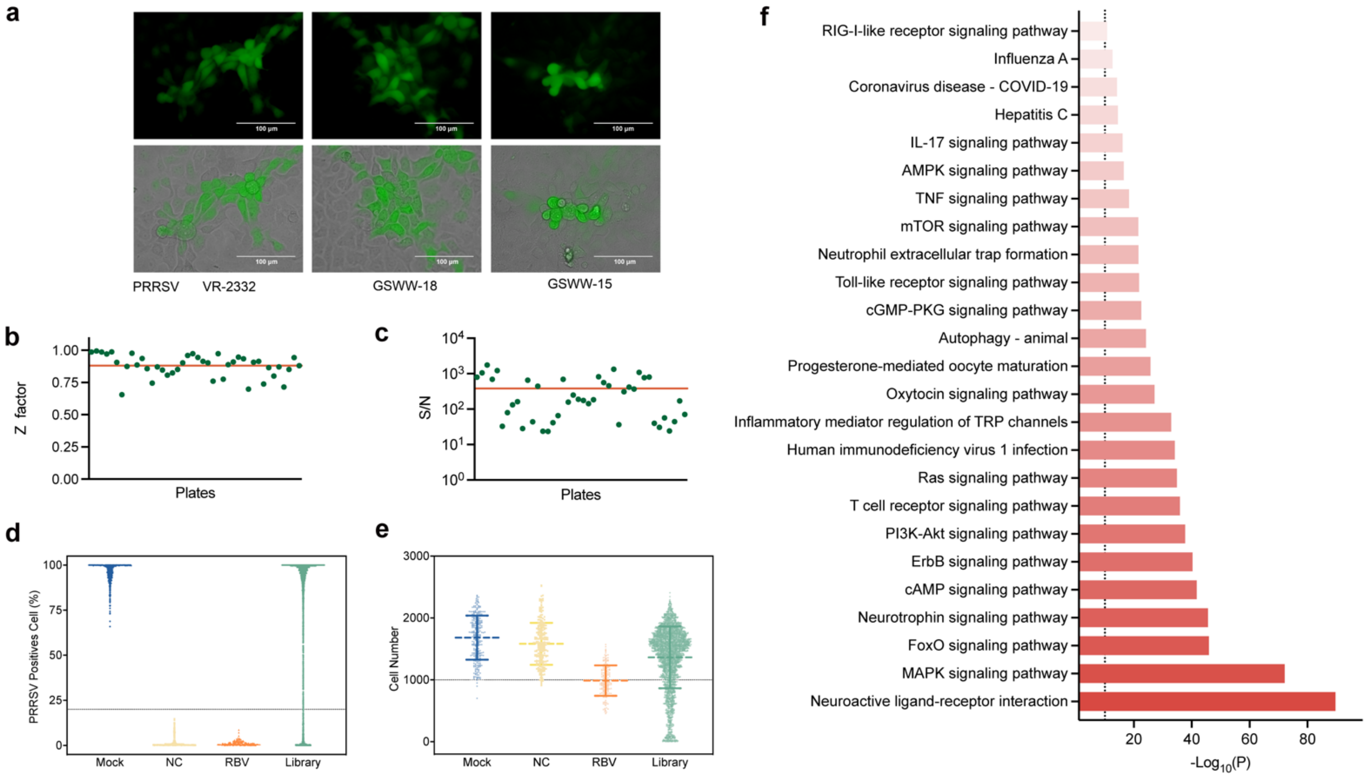

Supplementary Fig. 1: High-throughput screening for anti-PRRSV compounds.

(a) Cells infected with three different strains of PRRSV display green fluorescence.

(b) *Z′* factors for each plate among all screening plates (n = 42).

(c) Signal-to-noise ratios for each plate among all screening plates (n = 42).

(d) Positive rates of GFP-expressing cells categorized by different groups.

(e) Cell counts categorized by different groups.

(f) Enrichment analysis of target pathways for hit compounds. Analysis was conducted using the Metascape website, and p-values were calculated as described in Methods Enrichment Analysis.

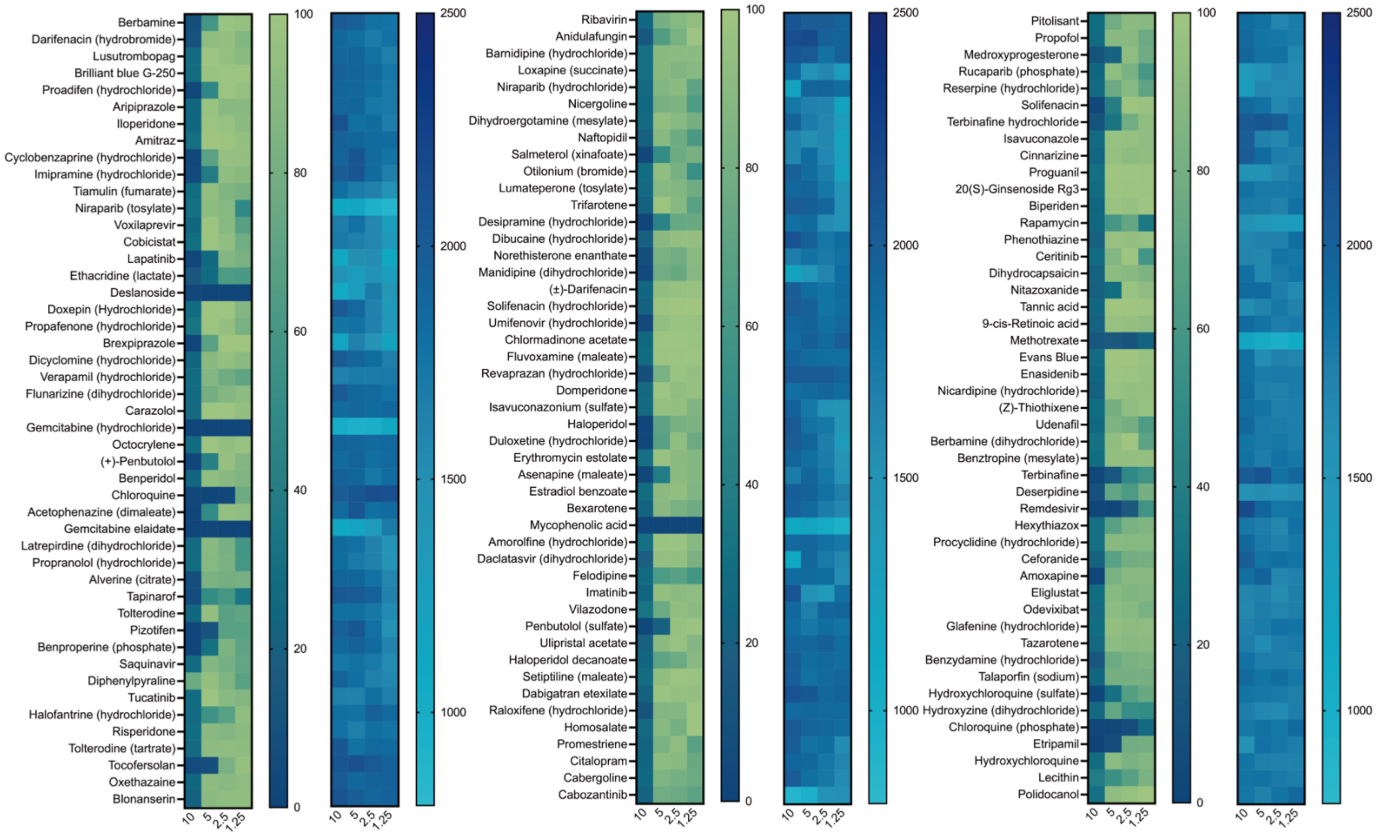

Supplementary Fig. 2: Dose Responses of 141 Compounds on Marc-145 Cells.

Compounds were initially diluted to 20 µM in cell culture medium, followed by two-fold serial dilutions, then co-incubated with Marc-145 cells and the GSWW-18 strain in a 96-well plate. The percentage of fluorescent cells and total cell counts were measured using the GE Cytell™ Cell Imaging System. The heatmap displays the percentage of fluorescent cells in green and total cell count in blue, with final compound concentrations at 10 µM, 5 µM, 2.5 µM, and 1.25 µM. Data represent averages from three to five images per well.

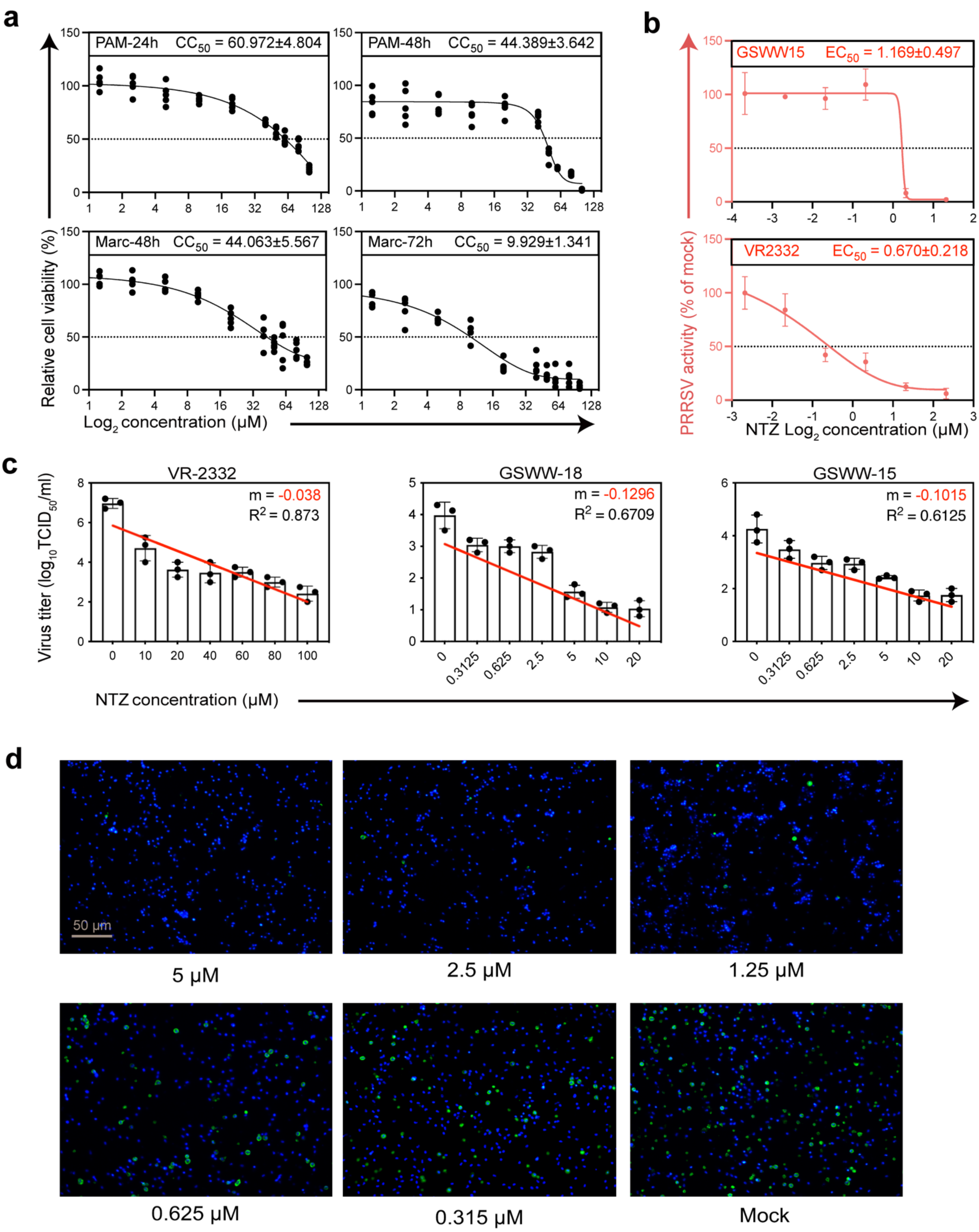

Supplementary Fig. 3: Cytotoxicity and Antiviral Efficacy of NTZ.

(a) CC_{50} of NTZ in various cell types determined using CCK-8 assay.

(b) EC_{50} of VR-2332 and GSWW-15 on PAM cells.

(c) Impact of NTZ on viral titers of VR-2332, GSWW-15, and GSWW-18 in Marc-145 cells.

(d) IFA results of the antiviral response against GSWW-18 strain in PAM cells treated with NTZ. PRRSV capsid protein positive (primary antibody SR30, green) and nuclei (DAPI, blue).

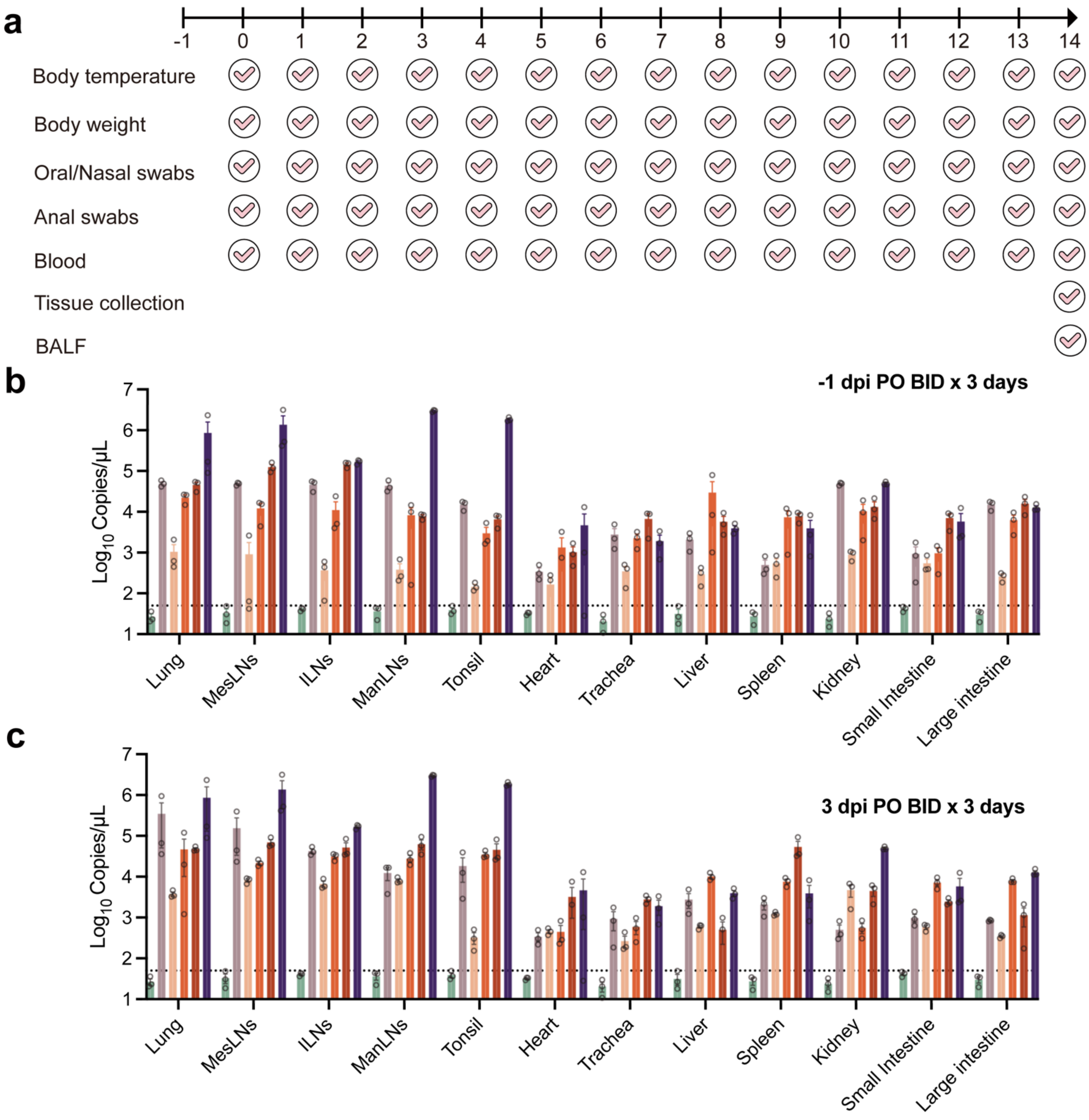

Supplementary Fig. 4: Viral Load in Various Tissues.

(a) Animal experimental timeline. Daily monitoring of animal body temperature and weight was conducted, with oronasal and anal swabs collected. Blood samples were drawn via anterior vena cava. Subsequently, as described in Method In Vivo Antiviral Effect of NTZ, one animal per group was randomly euthanized on day 14 for tissue and BALF collection.

(b and c) Viral loads in 12 different tissues from both prophylactic and treatment groups, represented by open circles indicating independent technical replicates. The dashed line denotes the limit of detection.

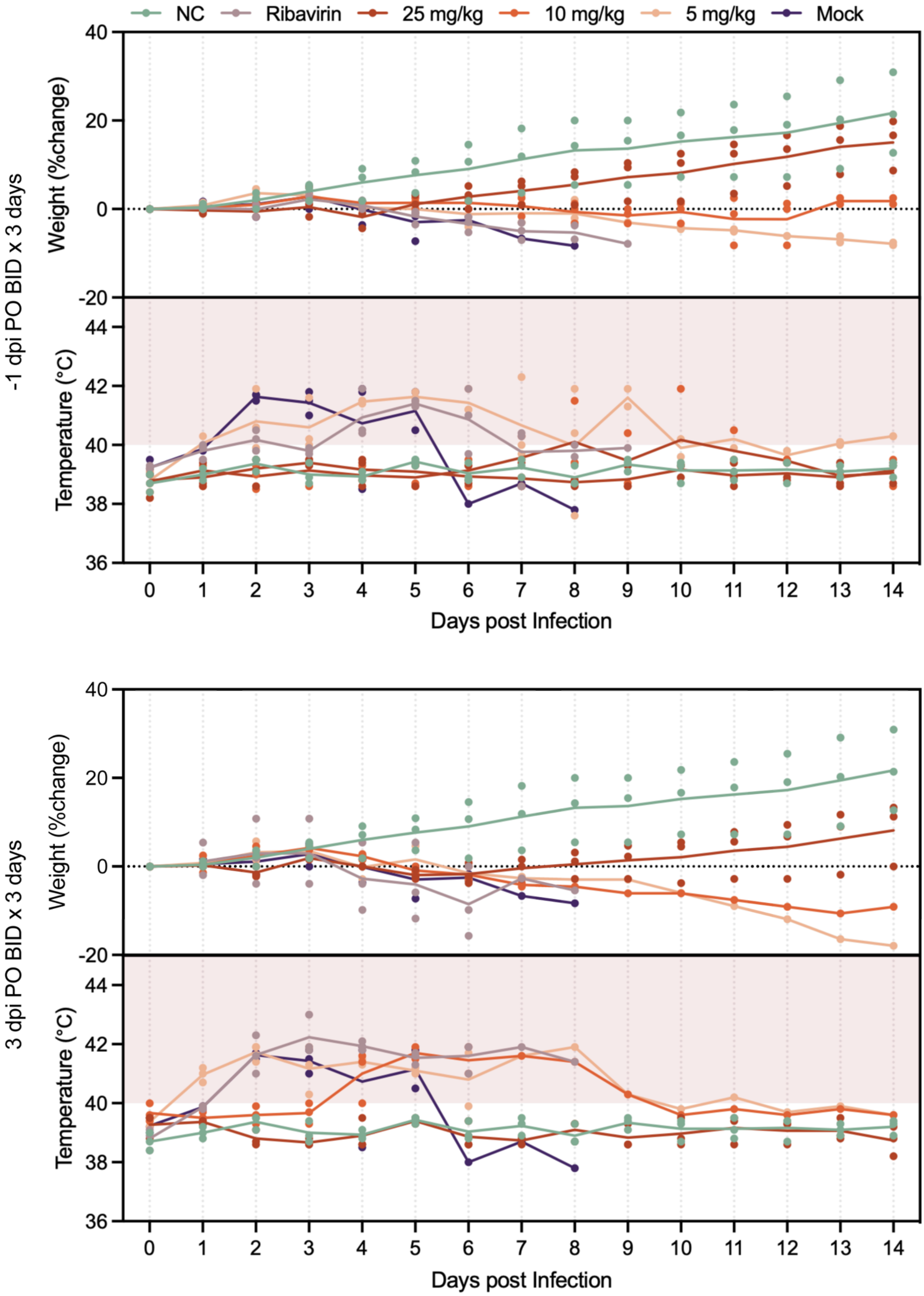

Supplementary Fig. 5: Changes in Body Temperature and Weight.

Daily monitoring of animals included rectal temperature and weight changes. The rate of weight change was calculated as described in Method In Vivo Antiviral Effect of NTZ. In the body temperature graphs, light red areas indicate temperatures exceeding 40°C, indicative of fever.

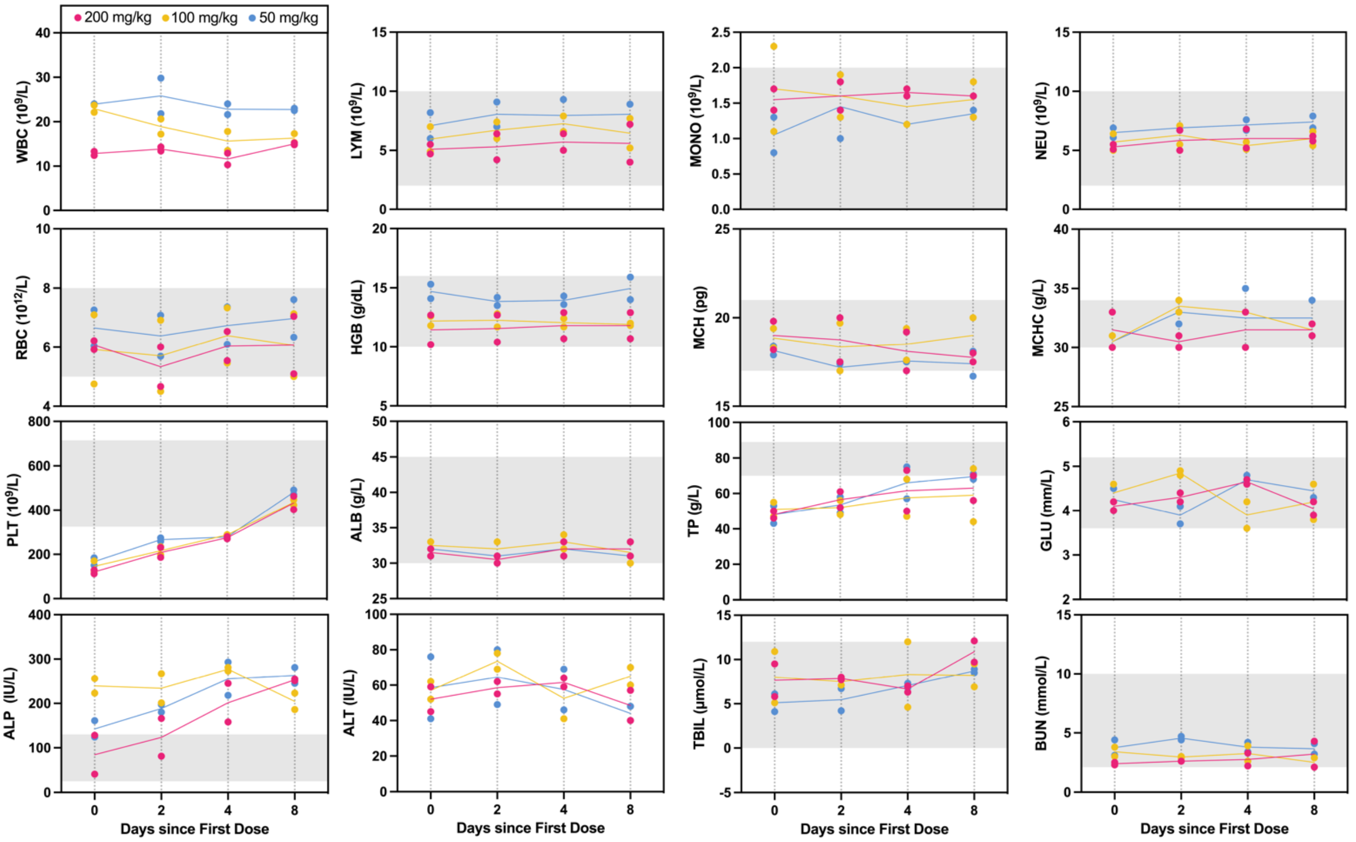

Supplementary Fig. 6: Complete Blood Counts and Biochemical Analysis.

Six animals from the same enclosure were divided into three groups (n = 2) and fed different doses of NTZ twice daily for three consecutive days, with a 12-hour interval. Parameters measured included White Blood Cell (WBC) counts, Lymphocyte (LYM) percentages, Monocyte (MONO) counts, Neutrophil (NEU) counts, Red Blood Cell (RBC) counts, Hemoglobin (HGB) concentration, Mean Corpuscular Hemoglobin (MCH), Mean Corpuscular Hemoglobin Concentration (MCHC), Platelet (PLT) counts, Albumin (ALB) concentration, Total Protein (TP) concentration, Glucose (GLU) levels, Alkaline Phosphatase (ALP) levels, Total Bilirubin (TBIL) levels, and Blood Urea Nitrogen (BUN) concentration. The grey areas in each panel represent the reference ranges for these indices.

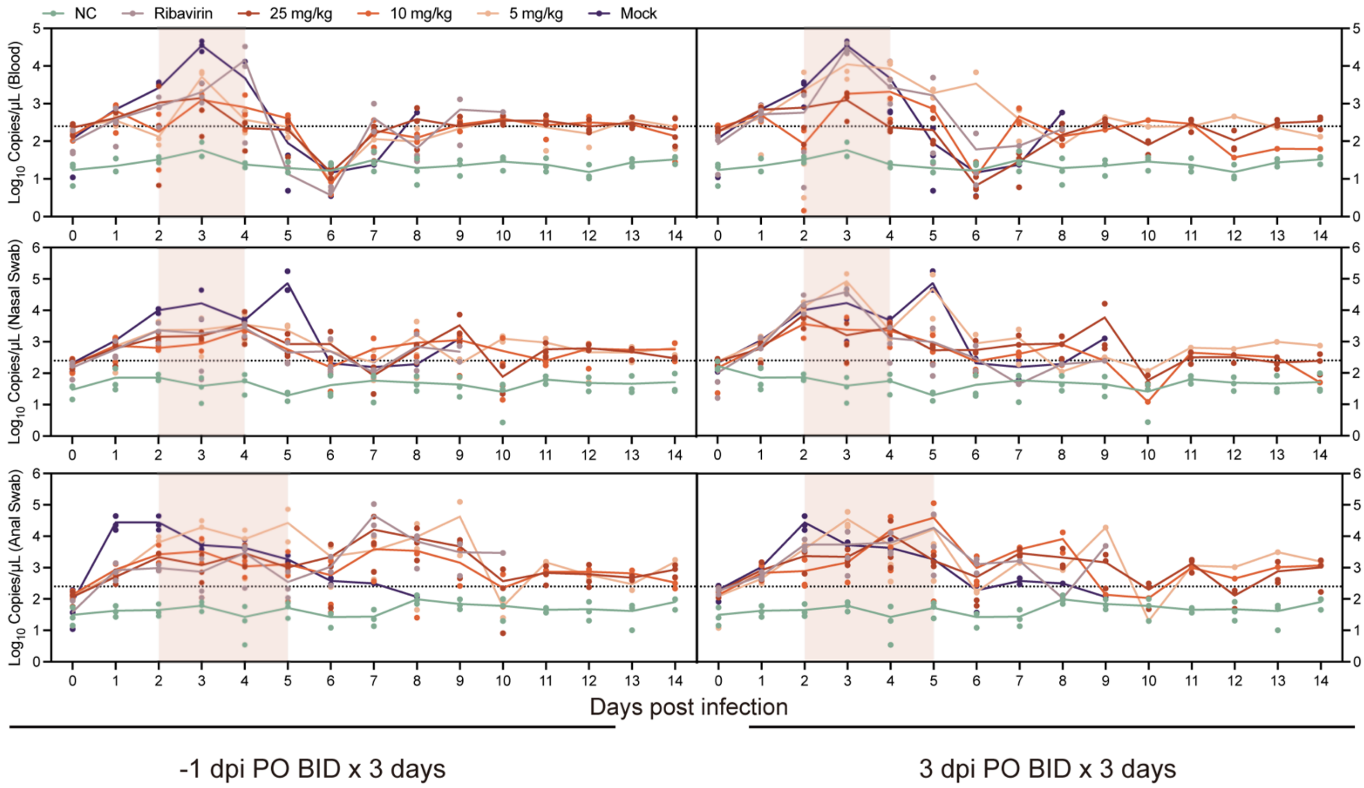

Supplementary Fig. 7: Viral Load in Blood, and Shedding in Oronasal and Anal Swabs.

Following our protocol **(Supplementary Fig. 4a)**, viral load in blood, and shedding in oronasal and anal swabs of all animals were monitored daily over a 14-day experimental period. Peak viral loads in blood and peak shedding in oronasal swabs were observed between 2 to 4 dpi, with peak shedding in anal swabs occurring between 2 to 5 dpi. Dashed lines in the line graphs represent the limit of detection.

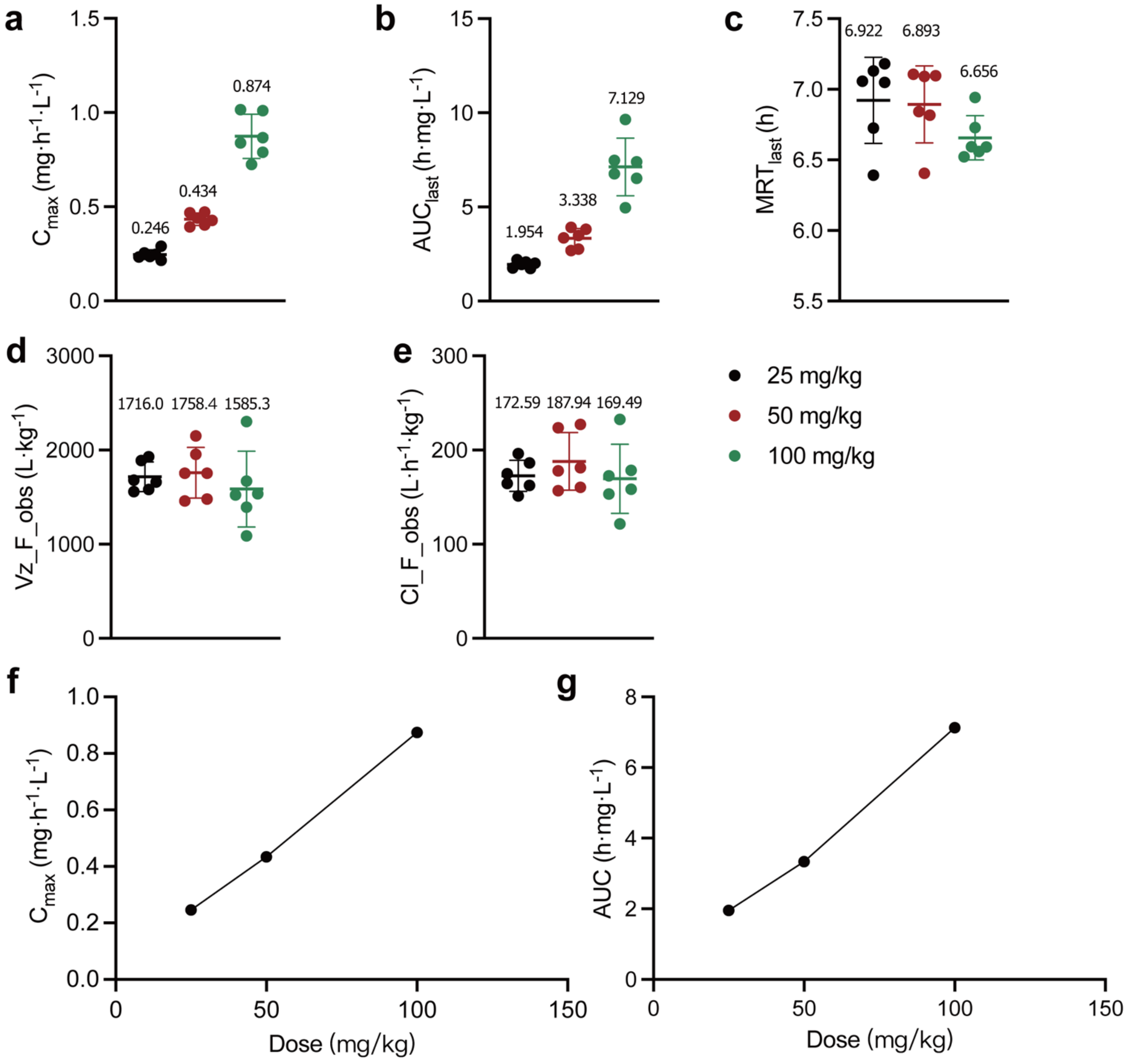

Supplementary Fig. 8: Pharmacokinetic Parameters.

As described in Method Pharmacokinetics and Tissue Distribution of NTZ, pharmacokinetic parameters were calculated using a non-compartmental analysis (NCA). Eighteen pigs were evenly divided into three dosage groups (*n* = 6) and housed separately. Fasting and water restrictions were implemented 12 hours prior to the experiment, with professional veterinary staff monitoring and cleaning enclosures to prevent coprophagia. Bars in the graph represent Mean ± SD.

(a) Peak drug concentration.

(b) Area under the curve from the time of dosing to the time of the last measurable (positive) concentration.

(c) Mean residence time from the time of dosing to the time of the last measurable concentration. **(d)** The Volume of distribution is based on the terminal phase.

(e) Total body clearance for extravascular administration.

(f and g) Linear relationship between oral dose and both C_{max} and AUC.

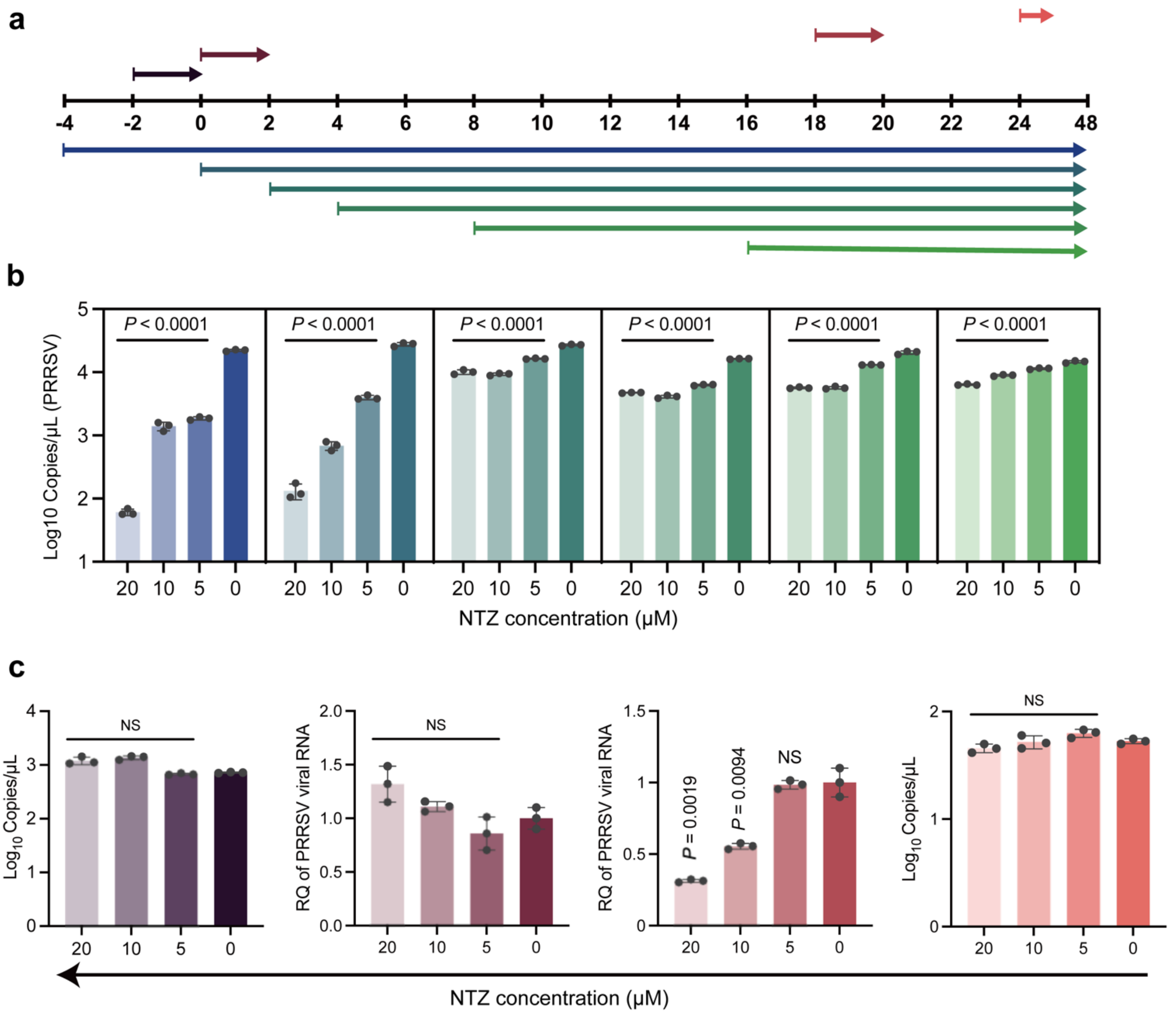

Supplementary Fig. 9: NTZ Inhibition of PRRSV Replication Stages.

(a) Schematic of the classic experimental approach of adding the drug at different time points. Cells were infected with VR-2332 (MOI = 1) at 0 h, followed by addition of NTZ at designated times.

(b) Different concentrations of the compound were added at -4, 0, 2, 4, 8, and 16 hours to observe its effect on various stages of viral replication.

(c) Viral inactivation (dark purple): PRRSV was pre-incubated with the compound for 1 hour in the incubator before transferring to monolayer Marc-145 cells, followed by medium change after 2 hours. Viral adsorption (light purple): Cells, virus, and compounds were placed at 4°C for 2 hours, then medium was refreshed. Viral replication: 18 hours post-infection, medium containing the compound was added, replaced by fresh medium after 2 hours. Viral release: 24 hours post-infection, medium containing the compound was added, replaced by fresh medium after 2 hours. All samples were collected and analyzed at 48 hours. Bar colors correspond to timing as shown in Fig. a. Symbols represent independent biological replicates **(b and c)**, with P-values as indicated. NS indicates $P > 0.0332$, calculated via two-way ANOVA with Dunnett's multiple-comparison test **(b and c)**; P-values < 0.0332 were considered significant.

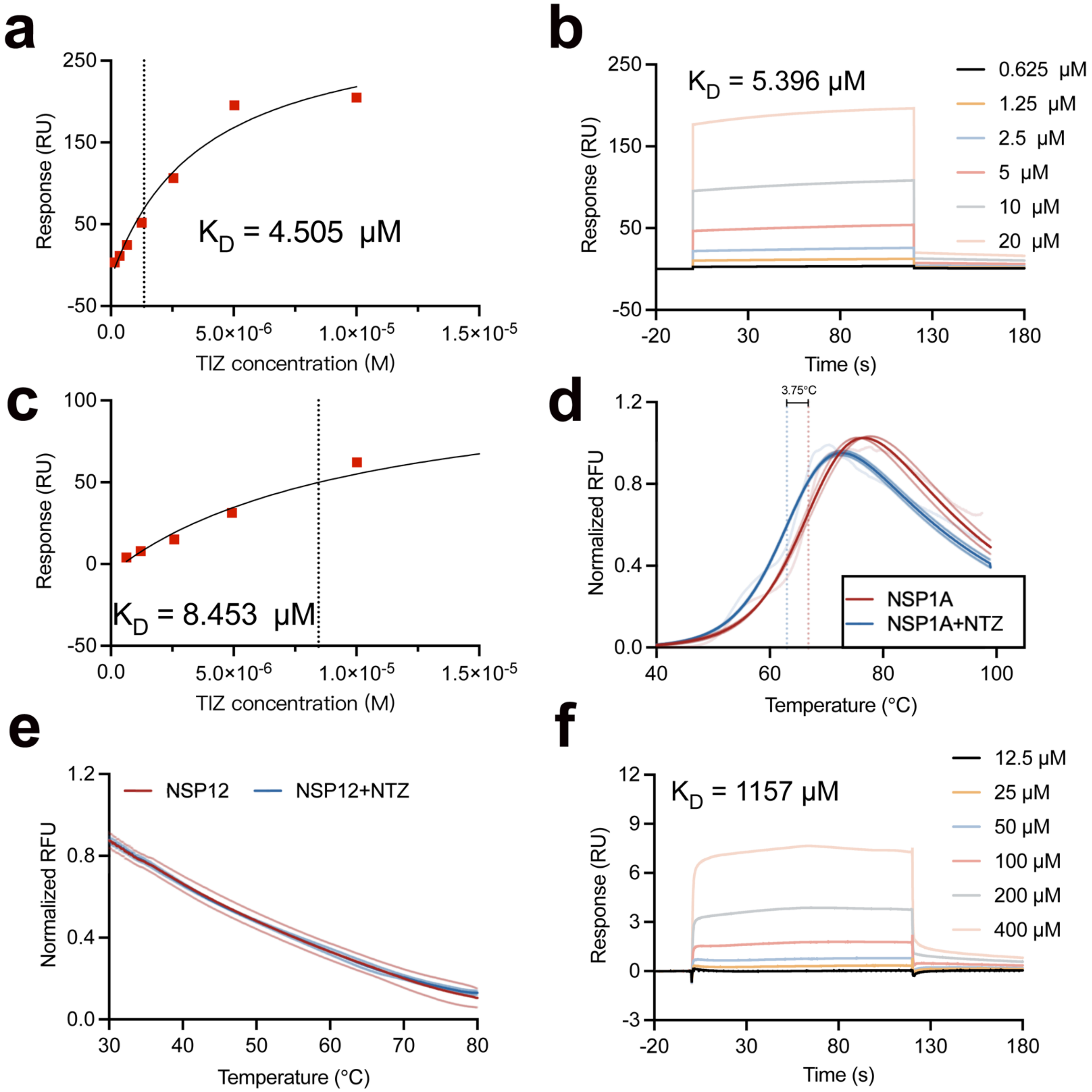

Supplementary Fig. 10: The Affinity of Nsp1α and Nsp12 with TIZ is Lower Than That of NMRAL1.

(a) The K_D value of NMRAL1 obtained through the Affinity fit model.

(b) The K_D value of Nsp1α determined using the Kinetics fit model.

(c) The K_D value of Nsp1α obtained through the Affinity fit model.

(d) DSF confirmed the interaction between in vitro purified Nsp1α and NTZ, resulting in a decrease of 3.75°C in the Tm value of Nsp1α.

(e) In the DSF, NSP12 purified by inclusion bodies (IBs) does not exhibit a Tm value.

(f) Through SPR, we found that TIZ only shows weak interaction with NSP12 protein purified in vitro.

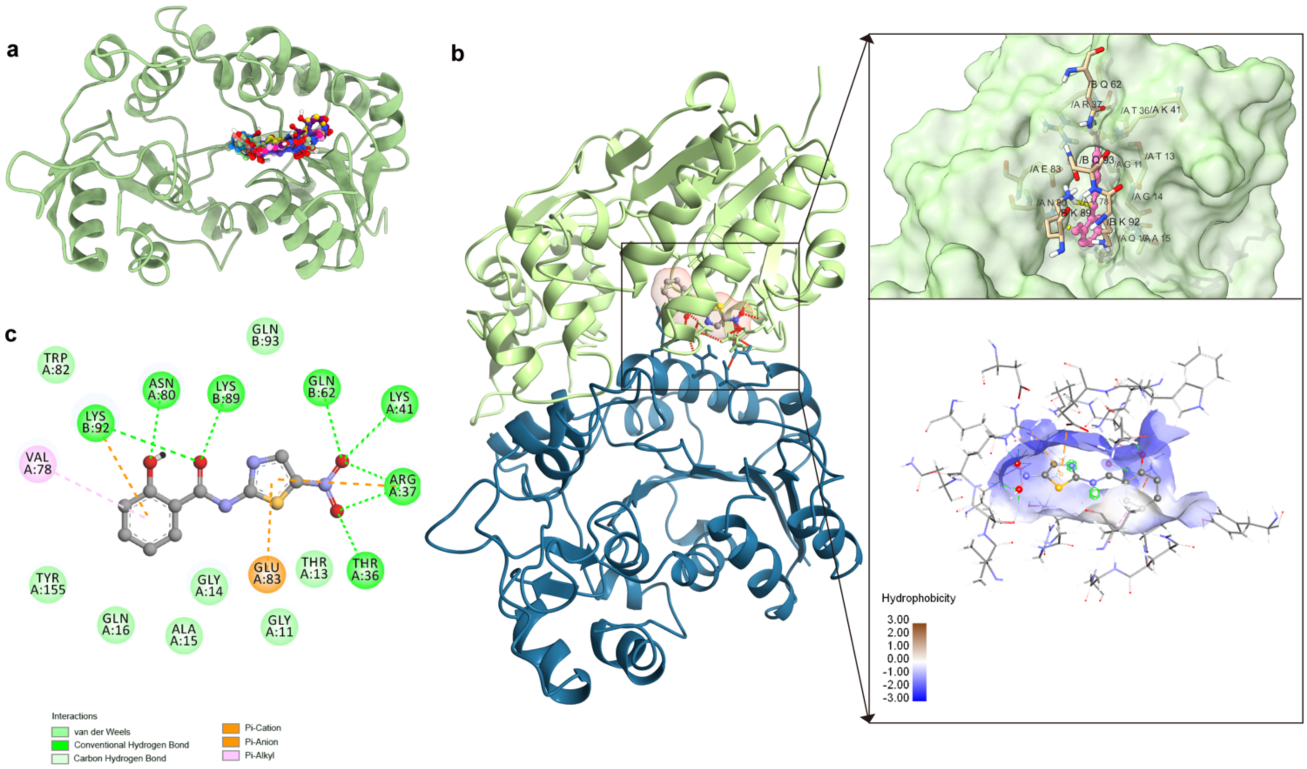

Supplementary Fig. 11: Molecular Docking of TIZ with NMRAL1 Dimer.

(a) Docking results of TIZ with NMRAL1 chain A. Position of TIZ (top 10 binding free energies).

(b, c) Interface interactions between NMRAL1 and TIZ. Seventeen amino acid residues of NMRAL1 are involved in interface interactions with TIZ. Binding primarily relies on hydrogen bonds, followed by Coulomb forces and hydrophobic interactions. For chain A (green), Thr36, ARG37, LYS41, ASN80, VAL78, and TYR155 form 12 hydrogen bonds with TIZ; while for chain B (blue), GLU62, LYS89, and LYS92 form 3 hydrogen bonds. Additionally, ARG37 and GLU83 in chain A and LYS92 in chain B exhibit electrostatic interactions with TIZ; VAL78 in chain A also shows hydrophobic interaction with TIZ **(Supplementary Table 2)**.

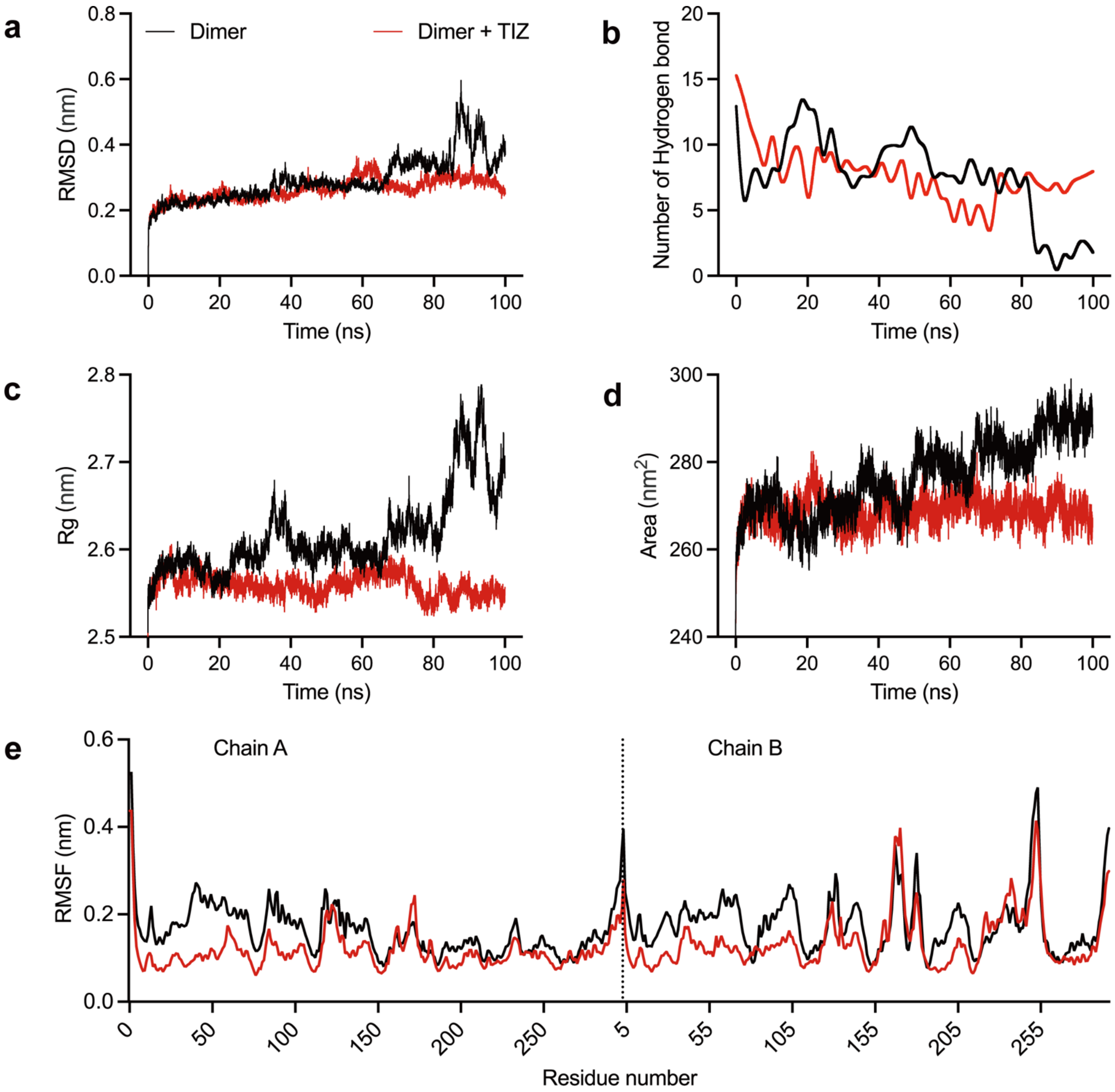

Supplementary Fig. 12: Molecular Dynamics Simulation Reveals TIZ Enhances Stability of NMRAL1 Dimer.

(a) Root-mean-square deviation (RMSD). In the absence of TIZ, RMSD (Cα) of the NMRAL1 Dimer increased after 80ns, indicating an increase in atomic distances.

(b) Number of hydrogen bonds. Without TIZ, the number of hydrogen bonds in the NMRAL1 Dimer decreased after 80ns, suggesting potential dissociation of chains A and B.

(c) Radius of Gyration (Rg). An increase in Rg indicates system expansion.

(d) Surface Solvent Accessibility (AREA). An increase in values suggests potential dimer dissociation, exposing previously shielded regions, hence increasing the area and indicating instability and spontaneous dissociation of the dimer during simulation.

(e) Root-mean-square fluctuation (RMSF). Overall, the RMSF values for Dimer + TIZ were lower than for Dimer alone, especially in the 30-60 region of chain A and the 85-105 region of chain B, where Dimer + TIZ showed a decrease in RMSF (∆rmsf > 0.2), possibly due to reduced fluctuations in these regions upon binding of NMRAL1 dimer and TIZ.

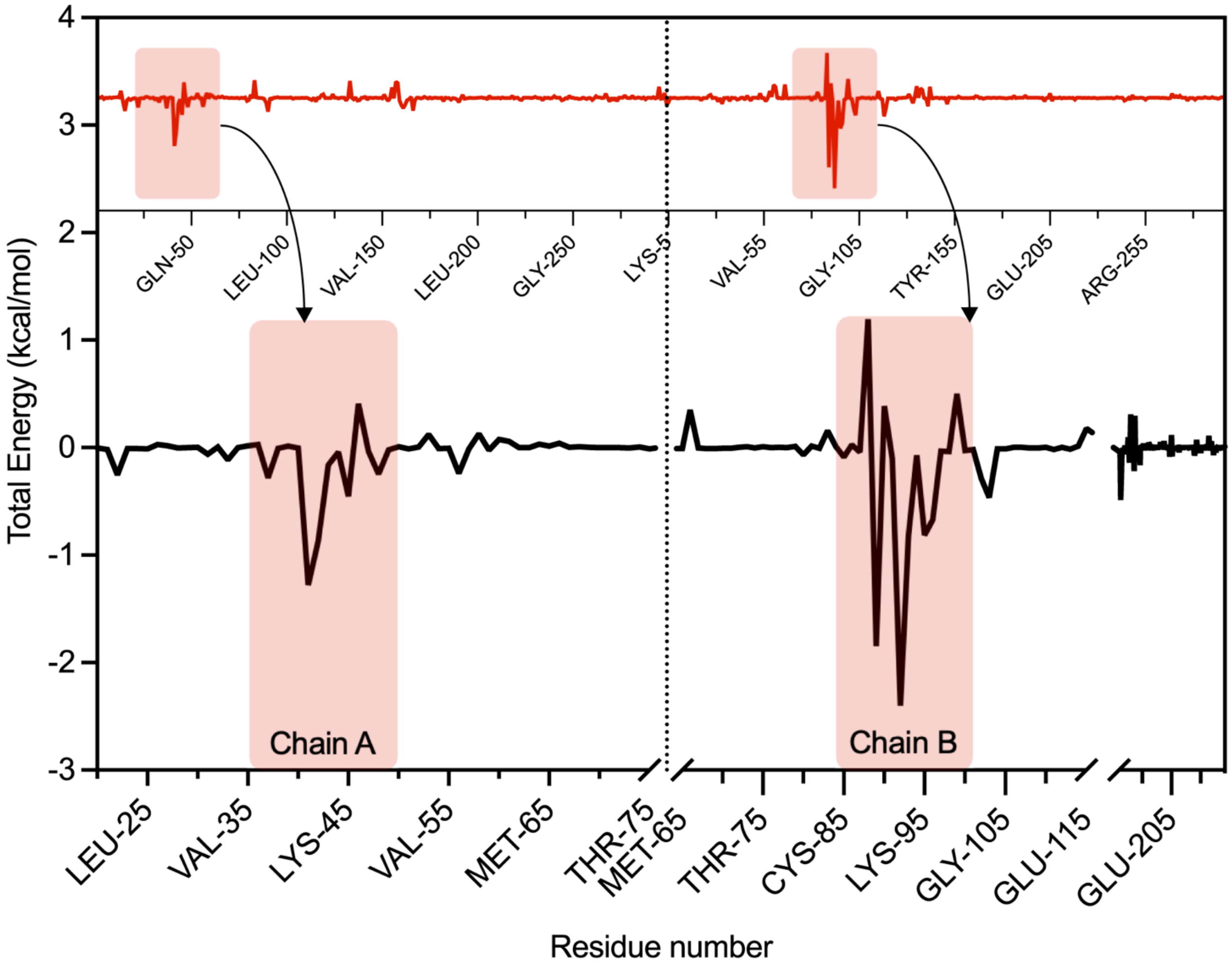

Supplementary Fig. 13: Energy Associated with Amino Acid Residues.

The binding free energy of TIZ with the NMRAL1 dimer is 70.543 kJ/mol **(Supplementary Table 3)**. TIZ primarily interacts with LYS41, LYS42, and LYS45 of chain A of the NMRAL1 dimer, and strongly with LYS92, LYS89, LYS95, GLN93, LEU96, LYS118, and ARG103 of chain B. Among these, LYS92 and LYS89 of chain B and LYS41 of chain A are hotspots, with binding energies greater than -1 kcal/mol **(Supplementary Table 4)**. This may explain the observed decrease in RMSF values in the 30-60 region of chain A and the 85-105 region of chain B, as shown in **Supplementary 12e**.

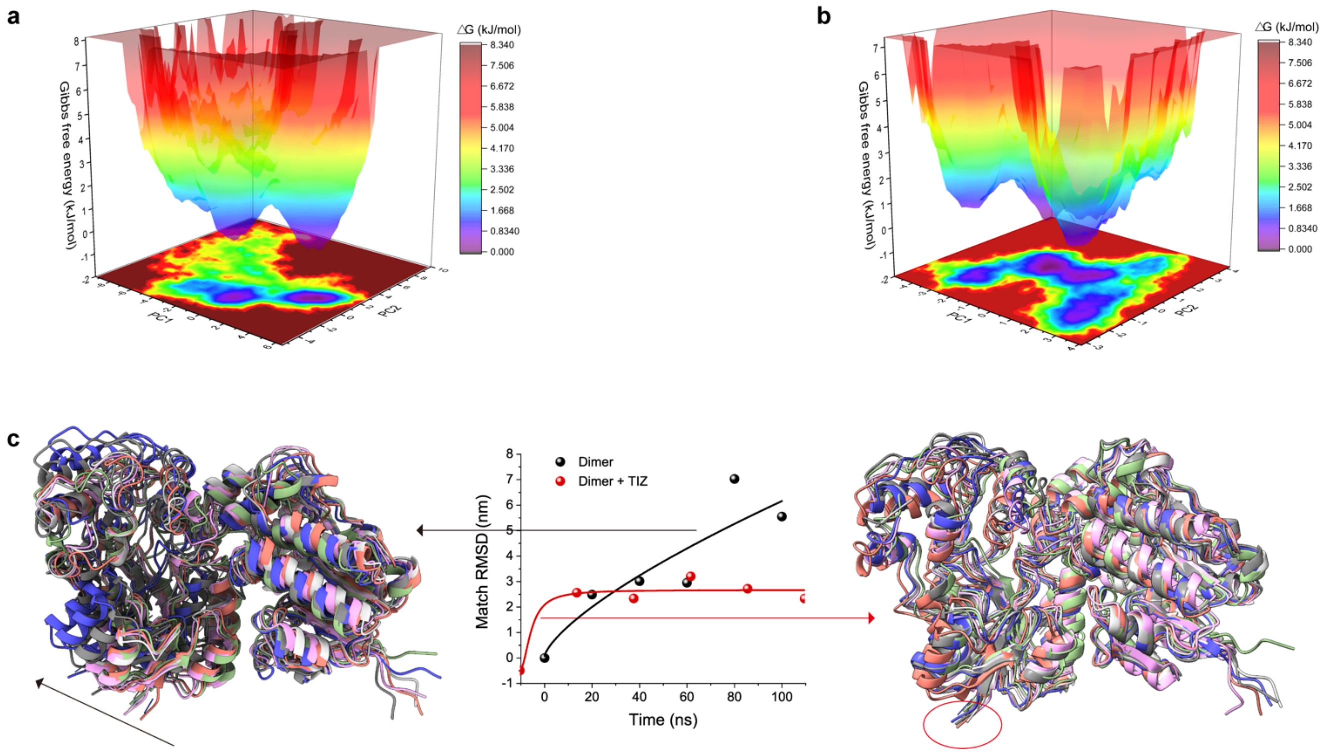

Supplementary Fig. 14: Changes in Protein Free Energy During Molecular Dynamics Simulation.

(a) Free Energy Landscape (FEL) of the NMRAL1 dimer without TIZ. **(b)** FEL of the NMRAL1 dimer with TIZ. Overall, the addition of TIZ results in reductions in both PC1 and PC2, with an overall lower energy, indicating a more compact structure of the NMRAL1 dimer upon TIZ binding. **(c)** Conformations of Dimer and Dimer+TIZ at 0, 20, 40, 60, 80, and 100 ns. For the NMRAL1 dimer without TIZ, the maximum match RMSD occurs at 80 ns with the greatest distance between chains A and B. The addition of TIZ does not result in a continuous increase in match RMSD. This phenomenon may be due to TIZ binding to the NMRAL1 dimer, linking chains A and B, and thus maintaining the dimeric state by restraining the chains A and B together during the simulation.

Supplementary Table 1 Pig health and clinical status scoring system.

Parameter	Standard	Fraction
Activity	Normal	0
	Slightly Reduced	1
	Stands Only When Forced	2
	Unable to Stand	3
Body Condition	Emaciated, Backbone and Ribs Visible	1
Cough	Mild	1
	Severe	2
Sneezing	Mild	1
	Severe	2
Respiration	Increased Frequency, Snoring, Chest Movement Barely Visible	1
	Increased Frequency, Snoring, Obvious Chest and Abdominal Movement	2
	Increased Frequency, Difficulty Breathing, Gasping, Open Mouth Breathing	3
Skin	Coat Erect	1
	Skin Discoloration, Red, Pale, Gray, or Yellow	2
	Skin Cyanotic	3
Ears	Ear Skin Discoloration, Red, Pale, Gray, or Yellow	1
	Ears Cyanotic	2
	Ear Necrosis	3
Eyes/Conjunctiva	Pink, Clear Discharge	1
	Inflamed Eyes, Cloudy or Swollen Discharge	2
	Severely Inflamed Eyes, Purulent Discharge, Vascular Engorgement	3
Nasal Discharge	Clear Discharge	1
	Thick Discharge	2
Appetite	Reduced Appetite	1
	Almost No Appetite	2
	No Appetite	3
Feces	Reduced Feces, Dry	1
	Feces with Little Fibrin Covering or Diarrhea	2
	No Feces, Rectal Mucosa, Watery or Bloody Diarrhea	3

Note: Body weight loss (%) = 100 × [(7 d weight)] − (0 d weight)]/[0 d weight].

Supplementary Table 2: Analysis of Interaction Forces Between NMRAL1 Dimer and TIZ Interface

Name	Distance	Category	Types	From	From chemistry	To	Chemistry
A: THR36: HG -TIZ: O	1.87	H-Bond	Conventional H-Bond	A: THR36: HG1	H-Donor	TIZ:O	H-Acceptor
A: ARG37: HN - TIZ: O	2.44	H-Bond	Conventional H-Bond	A: ARG37: HN	H-Donor	TIZ:O	H-Acceptor
A: ARG37: HE - TIZ: O	2.00	H-Bond	Conventional H-Bond	A: ARG37: HE	H-Donor	TIZ:O	H-Acceptor
A: ARG37: HH21 - TIZ:O	2.02	H-Bond	Conventional H-Bond	A: ARG37: HH21	H-Donor	TIZ:O	H-Acceptor
A: LYS41: HZ1 - TIZ: O	2.45	H-Bond	Conventional H-Bond	A: LYS41: HZ1	H-Donor	TIZ:O	H-Acceptor
A: LYS41: HZ2 - TIZ: O	2.74	H-Bond	Conventional H-Bond	A: LYS41: HZ2	H-Donor	TIZ:O	H-Acceptor
A: ASN80: HD21 - TIZ: O	1.97	H-Bond	Conventional H-Bond	A: ASN80: HD21	H-Donor	TIZ:O	H-Acceptor
B: GLN62: HE21 - TIZ: O	2.07	H-Bond	Conventional H-Bond	B: GLN62: HE21	H-Donor	TIZ:O	H-Acceptor
B: LYS89: HZ3 -TIZ: O	2.01	H-Bond	Conventional H-Bond	B: LYS89: HZ3	H-Donor	TIZ:O	H-Acceptor
B: LYS92: HZ3 - TIZ: O	2.94	H-Bond	Conventional H-Bond	B: LYS92:HZ3	H-Donor	TIZ:O	H-Acceptor
A: THR36: HA - TIZ: O	2.40	H-Bond	Carbon H-Bond	A: THR36: HA	H-Donor	TIZ:O	H-Acceptor
A: ARG37: NH2 - TIZ	4.12	H-Bond; Electrostatic	Pi-Cation; Pi-Donor H-Bond	A: ARG37:NH2	Positive; H-Donor	TIZ	Pi-Orbitals; Pi-Orbitals
B: LYS92: NZ - TIZ	3.95	Electrostatic	Pi-Cation	B: LYS92: NZ	Positive	TIZ	Pi-Orbitals
A: GLU83: OE2 - TIZ	3.85	Electrostatic	Pi-Anion	A: GLU83: OE2	Negative	TIZ	Pi-Orbitals
TIZ - A: VAL78	4.80	Hydrophobic	Pi-Alkyl	TIZ	Pi-Orbitals	A: VAL78	Alkyl

Supplementary Table 3: Binding Energy Analysis of NMRAL1 Dimer with TIZ

Contribution Type	Standard	Deviation (kJ/mol)
Van der Waals Energy	-92.632	±1.902
Electrostatic Energy	-34.832	±2.820
Polar Solvation Energy	68.211	±3.030
SASA Energy*	-11.170	±0.248
Binding Energy	-70.543	±1.622

*Note: SASA stands for Solvent Accessible Surface Area.

Supplementary Table 4: Residual Energy Decomposition

Chain	Residue Number	Molecular Mechanics (kcal/mol)	Polar Contributions (kcal/mol)	Apolar Contributions (kcal/mol)	Total Energy (kcal/mol)
B	LYS-92	-15.2919	6.1518	-0.9111	-2.40002
B	LYS-89	-8.8163	2.0888	-1.0074	-1.8471
A	LYS-41	-3.2398	-2.0907	-0.0791	-1.2789
A	LYS-42	-2.8636	-0.7499	0	-0.86605
B	LYS-95	-4.1681	0.7712	0	-0.81321
B	GLN-93	-3.8557	0.6808	-0.2201	-0.81033
B	LEU-96	-2.755	0.3674	-0.4259	-0.66979
B	LYS-118	2.1617	-4.2194	0	-0.48845
B	ARG-103	-1.9975	0.0499	0	-0.46519
A	LYS-45	-1.5029	-0.4004	0	-0.45281
