# Supplementary material for: High-throughput screening unveils nitazoxanide as a potent PRRSV inhibitor by targeting NMRAL1
Source: Nat Commun. 2024 Jun 6;15:4813. doi: 10.1038/s41467-024-48807-y (PMC11156899; doi:10.1038/s41467-024-48807-y)
Supplement: Supplementary file 3 — Description of Additional Supplementary Files [file 41467_2024_48807_MOESM3_ESM.pdf]

Description of Additional Supplementary Files

File Name: Supplementary Video 1

Description: This video visually presents the amino acid residues interacting with TIZ. Red dashed lines indicate hydrogen bonds within 3 Å of TIZ.

File Name: Supplementary Video 2

Description: This video shows the trajectory of NMRAL1 without TIZ. Over time, the distance between the two chains of NMRAL1 increases, indicating a trend toward dissociation.

File Name: Supplementary Video 3

Description: In this video, due to the presence of TIZ, the NMRAL1 dimer does not exhibit significant dissociation within a 100 ns timeframe.
