# Supplementary material for: High-throughput screening unveils nitazoxanide as a potent PRRSV inhibitor by targeting NMRAL1
Source: Nat Commun. 2024 Jun 6;15:4813. doi: 10.1038/s41467-024-48807-y (PMC11156899; doi:10.1038/s41467-024-48807-y)
Supplement: Supplementary file 7 — Reporting Summary [file 41467_2024_48807_MOESM7_ESM.pdf]

nature portfolio

Corresponding author(s): Zhanding Cui

Last updated by author(s): Apr 28, 2024

Reporting Summary

Nature Portfolio wishes to improve the reproducibility of the work that we publish. This form provides structure for consistency and transparency in reporting. For further information on Nature Portfolio policies, see our Editorial Policies and the Editorial Policy Checklist.

Statistics

For all statistical analyses, confirm that the following items are present in the figure legend, table legend, main text, or Methods section.

n/a	Confirmed	
☐	☒	The exact sample size (*n*) for each experimental group/condition, given as a discrete number and unit of measurement
☐	☒	A statement on whether measurements were taken from distinct samples or whether the same sample was measured repeatedly
☐	☒	The statistical test(s) used AND whether they are one- or two-sided<br>*Only common tests should be described solely by name; describe more complex techniques in the Methods section.*
☒	☐	A description of all covariates tested
☒	☐	A description of any assumptions or corrections, such as tests of normality and adjustment for multiple comparisons
☐	☒	A full description of the statistical parameters including central tendency (e.g. means) or other basic estimates (e.g. regression coefficient) AND variation (e.g. standard deviation) or associated estimates of uncertainty (e.g. confidence intervals)
☐	☒	For null hypothesis testing, the test statistic (e.g. *F*, *t*, *r*) with confidence intervals, effect sizes, degrees of freedom and *P* value noted<br>*Give P values as exact values whenever suitable.*
☒	☐	For Bayesian analysis, information on the choice of priors and Markov chain Monte Carlo settings
☒	☐	For hierarchical and complex designs, identification of the appropriate level for tests and full reporting of outcomes
☒	☐	Estimates of effect sizes (e.g. Cohen's *d*, Pearson's *r*), indicating how they were calculated

Our web collection on statistics for biologists contains articles on many of the points above.

Software and code

Policy information about availability of computer code

Data collection	No software was used
Data analysis	The following softwares and websites were used for the analyses in this study: GSEA 4.3.2, Graph Pad Prism 9.5.1, Metascape website (analysis ran in April 2020), Design & Analysis Software v2.6.0, Phoenix WinNonlin v8.2, Microsoft Excel v16.7, Spectronaut v16.0, SRplot, DSFworld, Biacore T200 Evaluation Software 3.0, Watvina, Gromacs-2023.2

For manuscripts utilizing custom algorithms or software that are central to the research but not yet described in published literature, software must be made available to editors and reviewers. We strongly encourage code deposition in a community repository (e.g. GitHub). See the Nature Portfolio guidelines for submitting code & software for further information.

Data

Policy information about availability of data

All manuscripts must include a data availability statement. This statement should provide the following information, where applicable:

- Accession codes, unique identifiers, or web links for publicly available datasets
- A description of any restrictions on data availability
- For clinical datasets or third party data, please ensure that the statement adheres to our policy

All data generated in this study are provided in the Supplementary Information/Source Data file. The MS raw files and proteome sequences data used in this study have been deposited to in the Proteome Xchange Consortium under accession code PXD050369, but the data are available when manuscript online. Additionally, the molecular dynamics trajectories data have been made publicly accessible via the public repository ScienceDB and are available here: https://10.57760/

sciencedb.18476.

Research involving human participants, their data, or biological material

Policy information about studies with human participants or human data. See also policy information about sex, gender (identity/presentation), and sexual orientation and race, ethnicity and racism.

Reporting on sex and gender	Not applicable
Reporting on race, ethnicity, or other socially relevant groupings	Not applicable
Population characteristics	Not applicable
Recruitment	Not applicable
Ethics oversight	Not applicable

Note that full information on the approval of the study protocol must also be provided in the manuscript.

Field-specific reporting

Please select the one below that is the best fit for your research. If you are not sure, read the appropriate sections before making your selection.

[x] Life sciences   [ ] Behavioural & social sciences   [ ] Ecological, evolutionary & environmental sciences

For a reference copy of the document with all sections, see nature.com/documents/nr-reporting-summary-flat.pdf

Life sciences study design

All studies must disclose on these points even when the disclosure is negative.

Sample size	We followed Jaykaran Charan's guidelines and used GPower to determine sample sizes39, dividing animals into 10 groups (n = 3).<br>For dose-responses, Marc-145 and PAM cells were used and n= 6 at least independent experiments were performed. In order to increase the statistical reliability for calculating EC50s (Sample size for other experiments specified in the figure legends).
Data exclusions	No data has been excluded from the analyses presented in this manuscript.
Replication	Detailed statistical information for each experiment is described in the figure legends and corresponding Extended Data.
Randomization	Groupings of animals were randomly selected.
Blinding	Data collection for the first screen and the validation in Marc-145-GFP was run blinded, without knowing which compound was spot in the specific wells. Data analysis was also blinded, with the i investigators only having internal IDs of compounds, without knowing their identity.

Reporting for specific materials, systems and methods

We require information from authors about some types of materials, experimental systems and methods used in many studies. Here, indicate whether each material, system or method listed is relevant to your study. If you are not sure if a list item applies to your research, read the appropriate section before selecting a response.

Materials & experimental systems

n/a	Involved in the study	
[ ]	[x]	Antibodies
[ ]	[x]	Eukaryotic cell lines
[x]	[ ]	Palaeontology and archaeology
[ ]	[x]	Animals and other organisms
[x]	[ ]	Clinical data
[x]	[ ]	Dual use research of concern
[x]	[ ]	Plants

Methods

n/a	Involved in the study	
[x]	[ ]	ChIP-seq
[ ]	[x]	Flow cytometry
[x]	[ ]	MRI-based neuroimaging

Antibodies

Antibodies used	The following antibodies were used for IFA and IHC: Mouse anti-SR30 (Rtilab, SR30-A), Goat Anti-Mouse HRP (Bioss, bs-0368G-HRP), Goat Anti-Mouse FITC (Bioss, bs-0368G-FITC).

	For protein immunoblotting: Rabbit anti-GATM antibody (HPA026077, Sigma), Rabbit anti-NMRAL1 antibody (K004351P, Solarbio), Mouse anti-HA antibody (26D11, Abmart), Mouse anti-GAPDH (60004, Proteintech), Goat Anti-Mouse HRP Conjugate (1705047, Bio-rad), Goat anti-Rabbit HRP Conjugate (1705046, Bio-rad).
Validation	Data are provided in the manuscript or refer to the manufacturer's website for information on antibody validation.

Eukaryotic cell lines

Policy information about cell lines and Sex and Gender in Research

Cell line source(s)	MARC-145 cells, a subclone of African green monkey kidney-derived MA-104 cells, were purchased from the China Center for Type Culture Collection(Wuhan, China). PAM cells were isolated and preserved from healthy pig lungs in our laboratory. The gender of the animals is randomized. Marc-145-GFP cells are the monoclonal cell line constructed and obtained in this work as described in the Methods and Materials section.
Authentication	All the cell lines were commercially available and have not been authenticated after receiving them.
Mycoplasma contamination	All cells tested negative for mycoplasma contamination.
Commonly misidentified lines (See ICLAC register)	No commonly misidentified cell lines were used in this study.

Animals and other research organisms

Policy information about studies involving animals; ARRIVE guidelines recommended for reporting animal research, and Sex and Gender in Research

Laboratory animals	All animal experiments were conducted according to protocols approved by the Lanzhou Veterinary Research Institute's Animal Ethics Committee. Pigs were housed in the institute's specific pathogen-free facility and cared for by professional veterinarians. They tested negative for ASFV, CSFV, PCV, and PRRSV via specific PCR/RT-PCR and commercial ELISA kits. All procedures conformed to China's general requirements for animal experiments (GB/T 35823-2018), and every effort was made to minimize animal discomfort.
Wild animals	N/A
Reporting on sex	The grouping of animals in this study was independent of sex.
Field-collected samples	Sample collection is carried out according to different experimental needs.
Ethics oversight	All animal experiments were conducted according to protocols approved by the Lanzhou Veterinary Research Institute's Animal Ethics Committee. All procedures conformed to China's general requirements for animal experiments (GB/T 35823-2018), and every effort was made to minimize animal discomfort. Euthanasia was administered to alleviate suffering in cases where animals couldn't feed or stand independently and showed diminished reactions to their environment.

Note that full information on the approval of the study protocol must also be provided in the manuscript.

Plants

Seed stocks	N/A
Novel plant genotypes	N/A
Authentication	N/A

Flow Cytometry

Plots

Confirm that:

- [ ] The axis labels state the marker and fluorochrome used (e.g. CD4-FITC).
- [x] The axis scales are clearly visible. Include numbers along axes only for bottom left plot of group (a 'group' is an analysis of identical markers).
- [x] All plots are contour plots with outliers or pseudocolor plots.
- [ ] A numerical value for number of cells or percentage (with statistics) is provided.

Methodology

Sample preparation	GFP fluorescence in Marc-145 or Marc-145-GFP cells, post PRRSV infection for 36 hours and subsequent trypsinization (0.25%, 25200072, Gibco).
Instrument	CytoFLEX LX (Beckman)
Software	FlowJo v10
Cell population abundance	N/A
Gating strategy	100% Marc-145 cell negativity is used as the limit for Marc-145-GFP cell negativity and positivity.

- [ ] Tick this box to confirm that a figure exemplifying the gating strategy is provided in the Supplementary Information.
